# The impracticality of radiomics research in acute ischemic stroke: from the perspective of primary healthcare institutions

**DOI:** 10.3389/fneur.2025.1559998

**Published:** 2025-09-17

**Authors:** Shaojun Zhang, Jiehao Tu

**Affiliations:** Department of Emergency, Shengzhou People’s Hospital (Shengzhou Branch of the First Affiliated Hospital of Zhejiang University School of Medicine, The Shengzhou Hospital of Shaoxing University), Shengzhou, Zhejiang, China

**Keywords:** acute ischemic stroke, radiomics, primary healthcare, practicality, predictive models

## Abstract

Acute ischemic stroke (AIS) is a leading global cause of disability and mortality, imposing a substantial socioeconomic burden. Neuroimaging serves as the primary and indispensable tool for AIS diagnosis and plays a pivotal role in guiding treatment decisions and prognostic evaluations. Radiomics enables the extraction of high-dimensional features from medical imaging data, which can be integrated with clinical endpoints to construct highly accurate predictive models, thereby informing disease diagnosis and therapeutic strategies. Consequently, radiomics-based investigations into AIS etiology, prognosis, and treatment selection have emerged as a prominent research focus. Numerous published studies have demonstrated that radiomics models achieve satisfactory predictive performance, offering valuable guidance across various clinical aspects of AIS. Primary care institutions represent the frontline in real-world AIS management—a critical yet often overlooked component of the diagnostic and therapeutic workflow. Their clinical capabilities significantly influence patient outcomes. Due to inherent resource limitations, these settings stand to benefit most from the translation of such research into practice. However, whether existing radiomics models are truly applicable to primary care remains unexplored. Thus, there is an urgent need for more radiomics studies tailored to the realities of primary care to address this gap. This article critically examines the potential limitations of current AIS radiomics research in terms of clinical utility for primary care settings and provides recommendations to guide future development and implementation.

## 1 Introduction

Stroke is the second leading cause of death globally and the third leading cause of disability (1). It is defined as an acute episode of focal dysfunction in the brain, retina, or spinal cord lasting more than 24 h—or any duration if neuroimaging (CT or MRI) or autopsy reveals focal infarction or hemorrhage consistent with clinical symptoms (1). Strokes are generally classified into two categories: hemorrhagic strokes (e.g., intracerebral hemorrhage) and ischemic strokes (e.g., cerebral infarction) (2). Acute ischemic stroke (AIS), accounting for approximately 80% of all strokes, results from inadequate cerebral blood flow, leading to ischemic hypoxia, localized tissue necrosis, or softening (3). Common causes of AIS include cerebral thrombosis, lacunar infarction, and cerebral embolism (3). Although stroke mortality has declined over the past two decades, the incidence of stroke, disability-adjusted life years (DALYs) lost, and the absolute number of stroke-related deaths continue to rise (4).

Radiomics is the process of converting digital medical images into high-dimensional data that can be mined for clinical insights (5, 6). By integrating imaging data with other patient information—such as clinical data, genomics, and drug responses—radiomics can significantly aid medical decision-making (7–10). Unlike qualitative assessments made by radiologists, radiomics transforms regions of interest (ROIs) within images into quantifiable, high-dimensional features, reducing subjective variability and improving diagnostic accuracy (11). Additionally, radiomics can help address disparities in imaging quality across hospitals and clinicians. Its versatility allows it to predict various clinical outcomes, including diagnosis, treatment plans, and prognosis, thereby supporting clinical decision-making (6). As a result, radiomics is increasingly applied in emergency medicine, assisting emergency physicians in the rapid diagnosis and treatment of AIS patients (12).

Neuroimaging, including cranial CT (Computed Tomography) and MRI (Magnetic Resonance Imaging), is essential in the management of AIS. It plays a critical role in diagnosing AIS, guiding treatment strategies, and assessing prognosis (13). In recent years, radiomics has been increasingly applied to AIS, with uses ranging from early diagnosis of stroke etiology to informing treatment decisions and predicting outcomes (14). For example, radiomics has been used to predict the likelihood of complications such as malignant brain edema, helping guide treatment choices, including conservative management or decompressive craniectomy (15, 16). Radiomics thus holds significant potential for improving AIS treatment and patient care.

Given the time-sensitive nature of AIS and the critical treatment window, patients are often transported to the nearest medical facility rather than directly to comprehensive stroke centers (17). While primary care institutions serve as the frontline for AIS management, limitations in diagnostic capabilities, imaging equipment, and treatment options frequently hinder accurate diagnosis and optimal therapeutic decision-making. Consequently, these settings stand to benefit most from clinically actionable radiomics research that could bridge existing gaps in AIS care. However, to our knowledge, despite the proliferation of radiomics studies in AIS, no research has systematically evaluated the applicability of these models from a primary care perspective. This paper examines the clinical practicality of existing AIS radiomics studies from the perspective of primary healthcare institutions, finding that most are unsuitable due to imaging modalities bias, clinical misalignment, implementation barriers, and performance gaps. This critique underscores the urgent need for radiomics research that addresses the realities of primary stroke care—where diagnostic uncertainty and time pressures are greatest. Future work must prioritize accessibility, interpretability, and immediate clinical actionability to fulfill the promise of precision medicine in AIS.

## 2 Image modality bias

**Figure 1:**
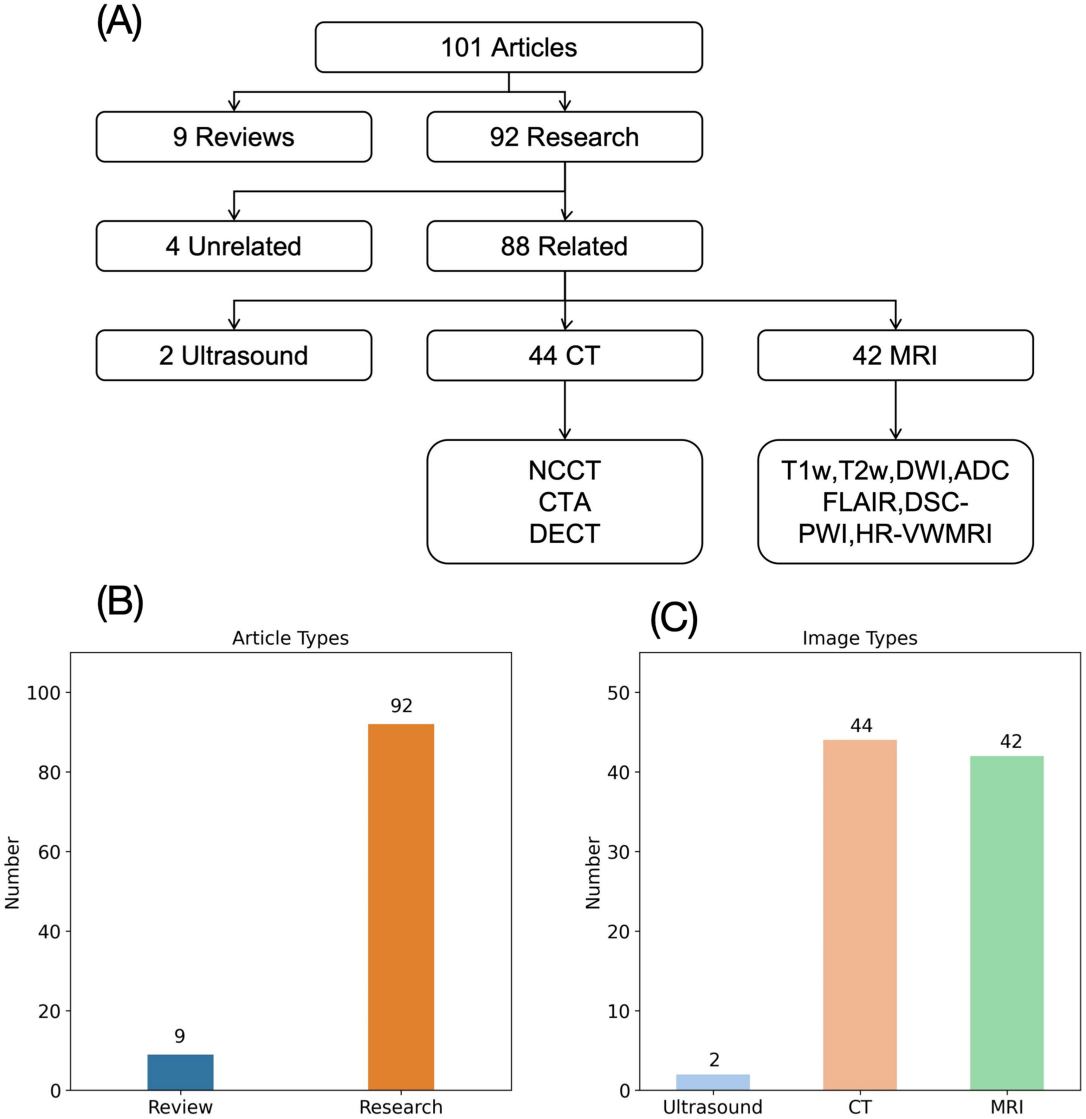
Image types of AIS radiomics research. **(A)** Flowchart of AIS radiomics image type analysis. **(B)** Types of literature on AIS radiomics research. **(C)** Distribution of imaging types of AIS studies.

We conducted a search on PubMed for articles published from January 2017 to November 2024 using the keywords “radiomics” and “AIS.” This search yielded 101 articles, including 9 reviews or meta-analyses and 92 research articles. Of the research articles, 4 were unrelated to radiomics or AIS, leaving 88 studies for analysis. These studies were categorized based on the imaging modalities used: CT, MRI, and ultrasound. Among the 88 studies, 44 utilized CT, 42 utilized MRI, and 2 utilized ultrasound (Figure 1). Of the 44 CT-based studies, 28 used NCCT (non-contrast CT), 11 used CTA (CT angiography), and 2 used both NCCT and CTA. The MRI-based studies included a variety of sequences, such as T1-weighted (T1w), T2-weighted (T2w), FLAIR (fluid-attenuated inversion recovery), DWI (diffusion-weighted imaging), ADC (apparent diffusion coefficient), as well as advanced sequences like DSC-PWI (dynamic susceptibility contrast perfusion-weighted imaging), HR-VWMRI (high-resolution vessel wall MRI), and HRMRI (high-resolution MRI) (Figure 1).

While acknowledging the undeniable value of MRI in the diagnosis and prognostic evaluation of AIS (18), we must recognize its limited suitability as a first-line imaging modality in clinical practice. The prolonged acquisition time necessitates prolonged patient immobility, which proves challenging for severe AIS patients experiencing delirium or agitation, often resulting in either non-compliance or significant discomfort. Furthermore, the MRI environment’s strict restrictions on metallic objects preclude the use of essential life-support equipment such as cardiac monitors, oxygen delivery systems, and infusion pumps—rendering the modality inaccessible for critically unstable patients. This inherent selection bias raises concerns about the generalizability of MRI-based radiomics research. Additionally, the substantial costs associated with MRI technology limit its availability primarily to urban tertiary care centers, while most AIS patients initially present to resource-limited primary care facilities due to time-sensitive treatment requirements (17). The financial impracticality of widespread MRI deployment in community hospitals fundamentally questions the clinical applicability of MRI-derived radiomics models in real-world settings where such infrastructure is absent. This significant disconnect between research focus and clinical reality is particularly evident given our finding that MRI-based studies constitute 42 out of 88 (47.7%) published AIS radiomics investigations.

Ultrasound is a convenient, cost-effective, and non-invasive diagnostic tool, but its use in diagnosing acute ischemic stroke (AIS) has not been well established. The two radiomics studies involving ultrasound and AIS reviewed in this paper primarily focus on analyzing carotid artery plaques to predict the risk of AIS (19, 20), rather than directly guiding its diagnosis or treatment. Currently, ultrasound is mainly used to assess large intracranial blood vessels (21), primarily for evaluating stenosis and vascular plaques. While significant occlusion or stenosis of large vessels can indicate ischemia or infarction in the corresponding regions, this connection is not always definitive. Moreover, intracranial vascular ultrasound is not a straightforward procedure, and many primary healthcare settings, such as emergency departments or ultrasound units, may lack the necessary equipment or expertise. Therefore, from a practical standpoint in primary healthcare institutions, radiomics research involving ultrasound for AIS diagnosis may have limited clinical value.

CT offers distinct advantages over MRI in the diagnosis of AIS. First, CT examinations are significantly faster than MRI—even when performing CTA or CTP, the acquisition time remains substantially shorter than DSC-PWI, markedly reducing patient cooperation requirements and minimizing discomfort. Importantly, the CT environment accommodates continuous cardiac monitoring and intravenous medication administration, significantly enhancing patient safety during scanning for severe AIS cases. From an infrastructure perspective, CT scanners are more affordable and therefore more widely available in primary care settings, which represent the frontline of AIS patient management. Clinically, since AIS symptoms (altered consciousness, motor/sensory deficits) closely resemble those of acute intracranial hemorrhage (22), CT serves as the primary modality for this critical differential diagnosis. The characteristic hyperdense appearance of hemorrhage versus hypodense ischemic changes on CT makes it the universal first-line imaging choice—virtually all suspected AIS patients undergo initial CT before subsequent MRI (when available) or treatment initiation (23). This clinical pathway ensures that CT images represent the largest potential dataset for radiomics research, where sample size directly correlates with model performance (24). For resource-limited primary care facilities that often can only perform CT, radiomics models derived from CT data could provide crucial decision support for diagnosis, treatment selection, and prognosis prediction. Despite this compelling rationale, our analysis revealed only 44 CT-based radiomics studies (50% of total publications), highlighting a critical need for more CT-focused AIS radiomics research that aligns with real-world clinical capabilities and workflows.

## 3 Prediction targets mismatch

**Figure 2:**
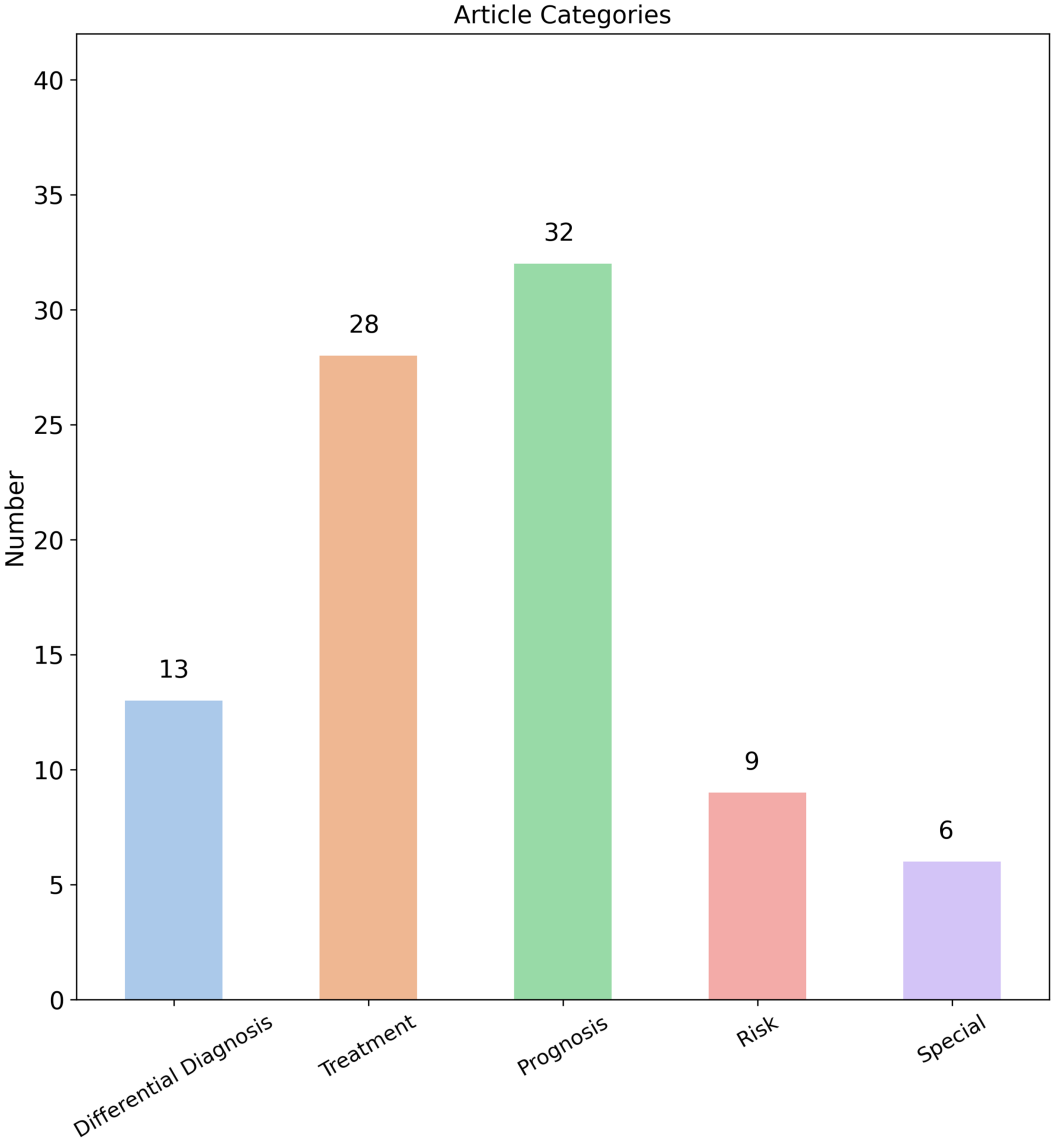
Distribution of prediction targets categories of AIS radiomics studies.

We analyzed the prediction targets of the 88 radiomics studies identified in our search and found that radiomics research on AIS covers a wide range of aspects, including diagnosis, treatment strategies, treatment outcomes, and prognosis. The breadth of these studies appears to be comprehensive. Based on the prediction targets of the radiomics models, we categorized the studies into five main groups: “Differential diagnosis,” “Treatment,” “Prognosis,” “Risk,” and “Special.” Among these, 32 studies focused on “Prognosis,” 28 on “Treatment,” 13 on “Differential diagnosis,” 9 on “Risk,” and 6 on “Special” (Figure 2). This distribution indicates that the primary focus of AIS radiomics research is on predicting prognosis and treatment outcomes.

We categorize disease progression, complications, and sequelae of AIS under the “Prognosis” category. Further analysis of these 32 prognostic studies revealed that 22 focused specifically on predicting functional outcomes in AIS patients, with the modified Rankin Scale (mRS) serving as the primary evaluation tool (25). Interestingly, 20 of the 22 studies on functional outcomes were based on MRI images. As mentioned earlier, MRI may not be readily available in primary healthcare settings, and not all AIS patients are suitable candidates for MRI. It must be acknowledged that in actual clinical practice at primary care institutions, most AIS patients are ultimately transferred to specialized stroke centers at tertiary hospitals. These advanced medical centers possess dedicated neurology and neuroradiology teams for comprehensive AIS management (26), where obtaining MRI examinations presents no significant difficulty. Under these circumstances, the application of MRI-based radiomics models for predicting functional outcomes in AIS patients represents an appropriate and feasible approach within such well-resourced settings. However, compared to functional outcomes in AIS patients, frontline physicians in primary care settings are more clinically concerned about other potential disease progression patterns, particularly hemorrhagic transformation and malignant cerebral edema. These complications typically indicate clinical deterioration, often necessitating urgent transfer to advanced medical centers or even emergency surgical operation (27, 28). For primary care facilities managing AIS cases, the ability to predict these hemorrhagic complications prospectively is critically important. A high-performance radiomics model capable of accurately predicting hemorrhagic transformation or malignant edema would enable risk stratification—guiding decisions about whether to administer tPA onsite or immediately transfer patients to tertiary hospitals. Expanding this concept further, radiomics could address other pressing clinical challenges in primary care, such as predicting risks of hemodynamic instability during transfer or recurrent infarction. These applications would directly solve real-world clinical dilemmas faced by community healthcare providers. Surprisingly, current radiomics research has largely overlooked these critical endpoints, with only 4 studies focusing on hemorrhagic transformation and 2 on cerebral edema among prognostic investigations—representing just a minimal fraction of the literature.

Treatment strategies, treatment outcomes, and treatment complications for acute AIS are grouped under the “Treatment” category, with a total of 28 studies. Rapid identification of cerebral infarction and prompt hospital transfer for thrombolytic therapy or mechanical thrombectomy can maximally reduce the incidence of disability (29–31). Therefore, as the first line of care for AIS patients, primary care physicians urgently need the capability to rapidly determine whether patients are eligible for thrombolysis or thrombectomy. The traditional paradigm of “time is brain” dictated that eligibility for thrombolysis or mechanical thrombectomy in AIS patients was determined solely based on whether they presented within the established treatment time window (32, 33). Since primary care facilities are typically only equipped to administer thrombolytic therapy, their physicians’ primary responsibility became assessing whether patients fell within the 4.5-h thrombolysis window—immediately initiating treatment for those meeting this criterion. However, in clinical practice, the actual stroke onset time is often uncertain due to various factors, creating significant challenges in determining whether patients remain within the therapeutic window and consequently affecting treatment decisions (34, 35). This clinical dilemma is reflected in our analysis, which identified 6 studies (out of 28 treatment-related publications) specifically focused on predicting stroke onset time. Notably, half of these (3 studies) utilized MRI-based radiomics models, again highlighting the tension between research approaches and real-world primary care capabilities where MRI is frequently unavailable.

However, the traditional time-window paradigm has significant limitations as it fails to account for individual patient variability. Current standards for mechanical thrombectomy in AIS have evolved from a time-centric approach to a tissue-based model, requiring comprehensive evaluation of both the ischemic penumbra and collateral circulation status in each patient (36, 37). Consequently, previous radiomics studies focusing solely on predicting time windows can no longer provide meaningful clinical guidance in isolation. Various imaging modalities, including CTP, CTA, DWI-PWI, and ASL (38), can effectively assess the ischemic penumbra. For primary care institutions, while DWI-PWI may be difficult to obtain, CTP represents the most accessible and practical option. However, despite its routine use in comprehensive stroke centers, CTP implementation in primary care settings still faces significant barriers, including high hardware and technical requirements, complex operation and interpretation protocols, as well as cost and insurance reimbursement limitations. The ideal scenario would involve overcoming these challenges to achieve widespread CTP adoption across primary care facilities. For institutions where CTP remains unavailable, an alternative interim solution could involve radiomics models capable of accurately predicting penumbra-to-core infarction ratios using NCCT alone—this would similarly enable clinicians to make informed treatment decisions. However, no such radiomics models have been reported in current literature.

For AIS patients eligible for either thrombolysis or endovascular therapy, treatment efficacy and potential complications naturally become the primary focus (39). Our analysis identified 21 relevant studies, with the majority (19 papers) being CT-based radiomics research focused on predicting outcomes and complications of endovascular therapy. These endovascular interventions—including intra-arterial thrombolysis, mechanical thrombectomy, and emergency angioplasty—require specialized DSA equipment, interventional devices, and neurointerventional specialists (40). For primary care institutions, establishing such comprehensive capabilities presents even greater challenges than acquiring MRI technology, creating a significant disconnect between current radiomics research focus and frontline clinical realities. Although endovascular therapy is primarily performed at specialized comprehensive stroke centers, the ability to predict treatment outcomes and complications using NCCT—readily available in primary care settings—could significantly improve patient management. Promising progress has already been made in this area. Wen et al. developed a radiomics model based on pre-intervention NCCT in acute anterior circulation infarction patients that accurately predicted post-thrombectomy malignant cerebral edema with an AUC reaching 0.879 (41). When advanced stroke centers are forewarned of high malignant edema risk through such predictions, they can better inform families during preoperative consultations, prepare for potential decompressive craniectomy during the procedure, and intensify postoperative osmotic therapy (e.g., mannitol administration). In another advancement, Hofmeister et al. utilized radiomic features of thrombi on pre-procedural NCCT to predict both first-pass successful recanalization with the ADAPT technique and the likely number of retrieval attempts needed if stent-assisted thrombectomy becomes necessary (42). These predictive insights provide valuable guidance for neurointerventionalists in selecting optimal endovascular strategies. While thrombectomy remains beyond their capabilities, primary care institutions can still administer intravenous thrombolysis for AIS patients. However, notably, our analysis identified only two radiomics studies focusing on intravenous thrombolysis in AIS patients. Among these, one developed its prediction model using DSC-PWI—an advanced MRI perfusion technique typically unavailable in primary care settings (43). The sole remaining study utilized NCCT and CTA imaging to predict the likelihood of successful recanalization following alteplase administration (44). Consequently, radiomics models predicting post-thrombolysis outcomes—including recanalization success, hemorrhagic transformation, or cerebral edema—would better align with the actual clinical needs of primary care institutions compared to thrombectomy recanalization prediction models (45). This targeted approach would directly address the decision-making challenges faced by frontline physicians managing AIS patients within their therapeutic capabilities.

The “Differential diagnosis” category includes only 13 studies, yet 7 of these focus on carotid artery plaques and thrombosis. For example, some radiomics models aim to predict the vulnerability of carotid artery plaques or the physiological and compositional properties of thrombosis (46–49). Clearly, these prediction targets are more aligned with pre-stroke risk assessment rather than directly addressing the diagnosis and treatment of AIS, which is the primary focus of this paper. Similarly, the 9 studies categorized under “Risk” all focus on predicting the likelihood of cerebral infarction, which again does not match the objectives of this paper. The “Special” category includes studies on the stability of radiomics features (50), such as the conversion of CT to MRI (51), which are also not directly relevant to the diagnosis and treatment of AIS.

In conclusion, while radiomics research on AIS has broadly addressed various aspects such as diagnosis (52), treatment strategies (42), treatment outcomes (53, 54), and prognosis (55, 56), few studies offer genuine clinical utility for primary care settings. Some studies focus on prediction targets that do not align with the capabilities of primary healthcare facilities, while others address issues that are not directly relevant to their clinical priorities. This disconnect likely stems from the academic origins of most radiomics research, as teams from tertiary medical centers naturally focus on challenges relevant to their advanced practice environment rather than primary care needs. It’s easy to add to abundance, but hard to relieve necessity. Future AIS radiomics research would benefit greatly from incorporating primary care perspectives—aligning prediction targets with actual diagnostic dilemmas and therapeutic decision points faced by community physicians. Only through such targeted approaches can radiomics truly address the most pressing needs at the frontline of stroke care.

## 4 Prediction models not instrumentalized

**Figure 3:**
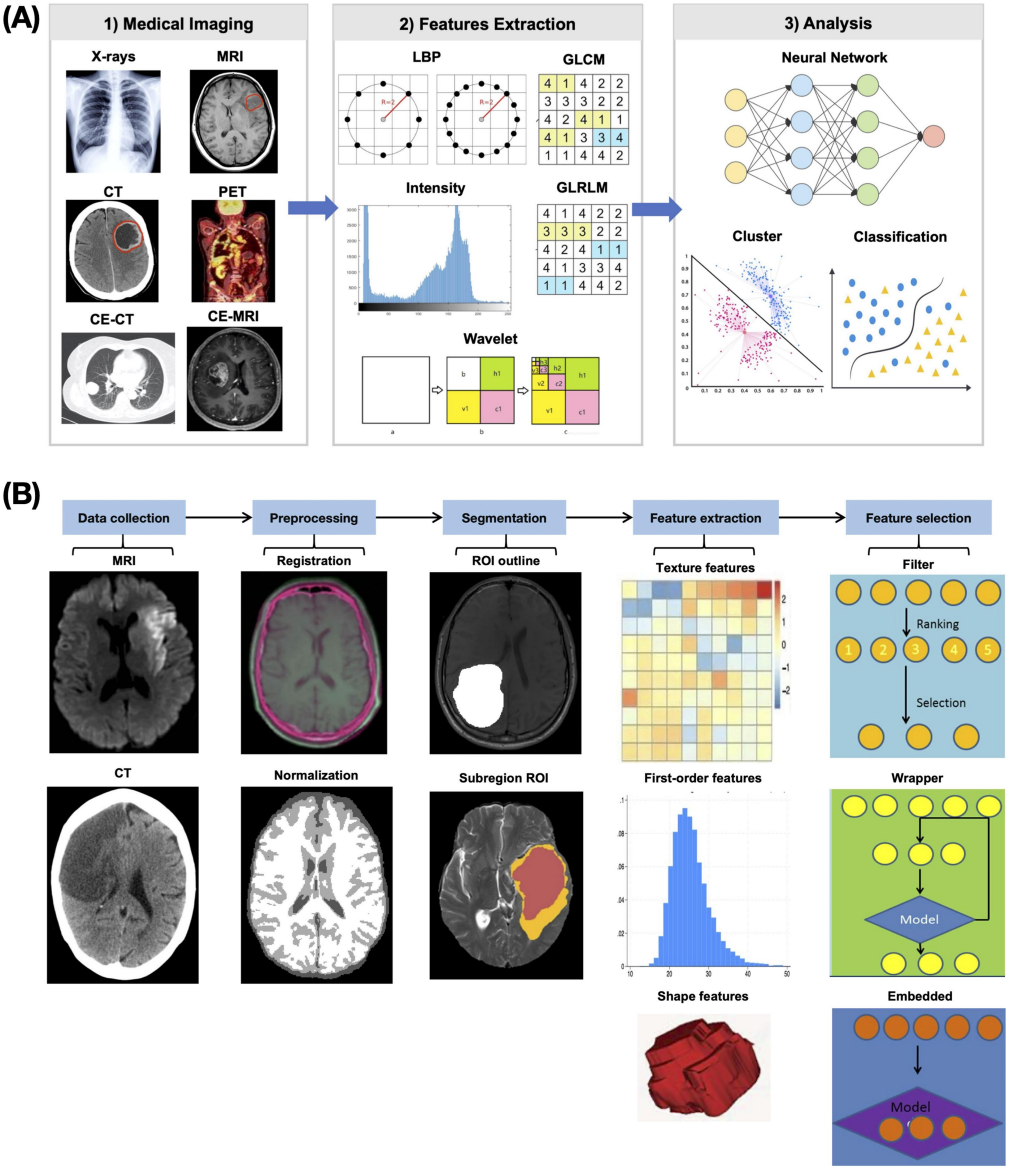
Flowchart of radiomics. **(A)** Schematic diagram of radiomics research. Radiomics research is the process of extracting radiomics features from medical images and then using them to build models to achieve various goals. **(B)** Schematic diagram of the radiomics feature extraction process. Through a series of image preprocessing steps, comparable radiomics features that can reflect image characteristics are extracted.

Radiomics represents a computational process that converts medical images into analyzable high-dimensional data through standardized procedures including image acquisition, preprocessing, feature extraction, and predictive modeling (11) (Figure 3). This methodology integrates expertise from statistics, programming, and machine learning, typically requiring multidisciplinary team collaboration due to its technical complexity (57). The ultimate objective is to develop clinically applicable predictive models using quantitative imaging features for specific clinical endpoints. In theory, consistent application of published radiomics protocols to AIS patient CT or MRI scans should yield comparable predictive results to guide clinical decisions. However, from the perspective of primary healthcare institutions, the key question remains: Are radiomics-based predictive models for AIS truly practical and useful in real-world clinical practice?

**Figure 4:**
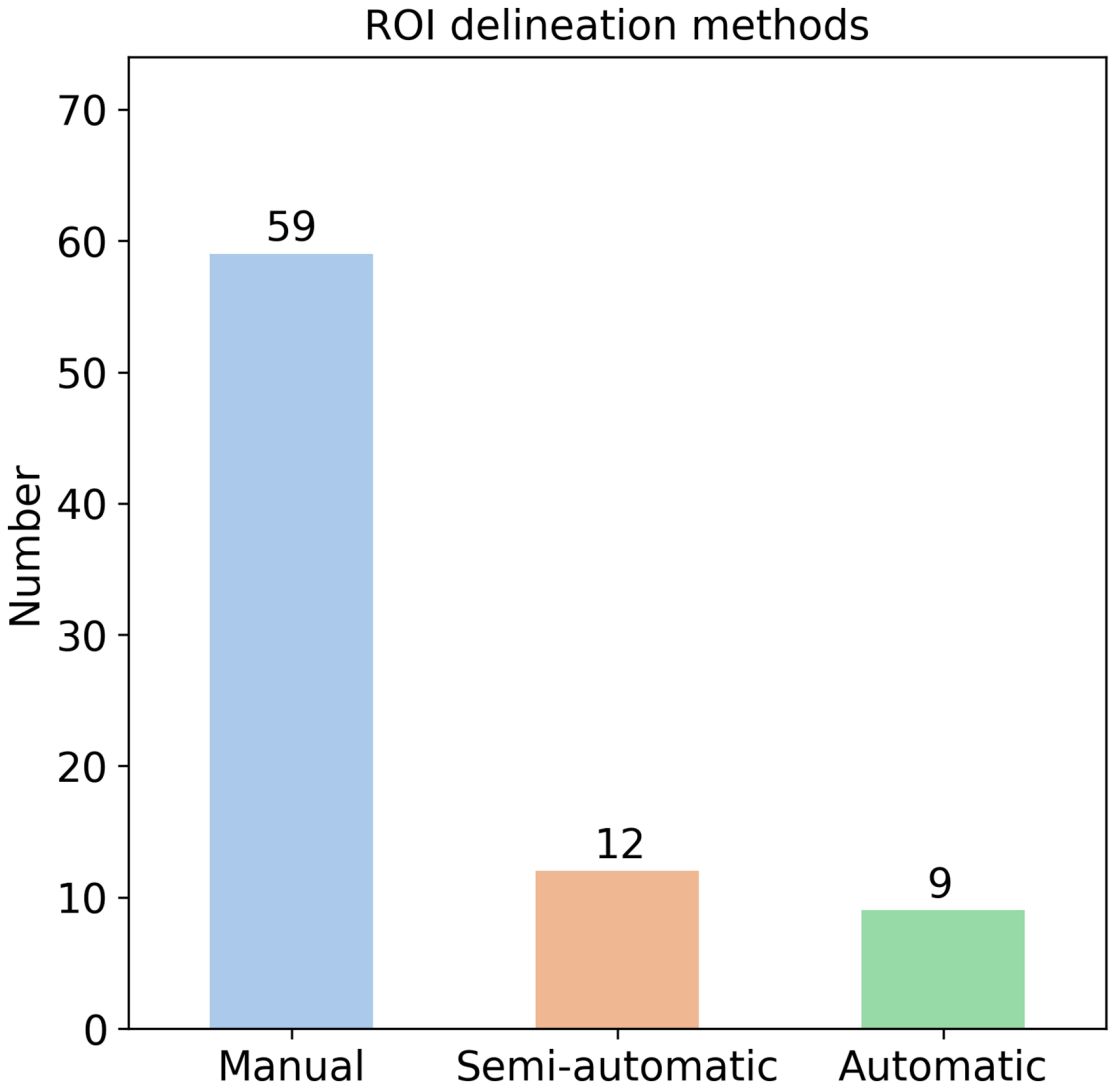
Region of interest delineation methods of AIS radiomics studies.

It is well-established that radiomics features are extracted from ROIs in medical images (11), with three primary segmentation methods employed: manual, semi-automatic, and automatic. Manual segmentation typically involves multiple radiologists independently delineating ROIs (e.g., hemorrhage or infarction areas) slice-by-slice to create precise masks (58). Semi-automatic approaches utilize threshold-based techniques for ROI identification (59), while automatic methods leverage specialized software or neural networks for segmentation (60). In our analysis of 82 AIS radiomics studies, we found that 59 studies (72%) used manual delineation, 12 studies (15%) used semi-automatic methods, and 9 studies (11%) used automatic methods (Figure 4). Additionally, two studies used the entire brain as the ROI.

Despite the prevalence of manual delineation, this method has several significant drawbacks. First, the labor-intensive process typically requires experienced radiologists to meticulously delineate ROIs slice-by-slice (58), particularly challenging for thin-section imaging like CTA or DSC-PWI where slice thickness often measures <1 mm, potentially necessitating annotation across dozens of slices. This time-consuming process (often requiring >15 min per case) becomes particularly problematic for time-sensitive AIS management, where every minute counts in preserving brain tissue. This paradox is starkly illustrated in time-window prediction studies, where 5 of 6 identified publications relied on manual segmentation despite their purported goal of accelerating treatment decisions (61–65), and 1 used a semi-automatic threshold-based method (66).

Second, manual segmentation suffers from substantial inter-rater variability, with different radiologists often producing markedly different ROI boundaries that may significantly impact extracted features and subsequent predictions (67). Nearly all manual segmentation studies involve ≥2 experienced radiologists, frequently requiring third-party adjudication for discordant cases—a luxury unavailable in many primary care settings, especially during off-hours when AIS frequently occurs. Compounding these challenges, ischemic lesion boundaries on CT are notoriously ambiguous, potentially magnifying interpretation discrepancies (68). The clinical stakes are high: inaccurate predictions could directly compromise patient outcomes. While automated/semi-automated methods (neural networks in 6 of 9 automated studies) theoretically address these inconsistencies, they introduce their own barriers: substantial hardware requirements and technical expertise that may exceed primary care capabilities (60, 69, 70). This creates a fundamental implementation gap between radiomics research and frontline clinical realities.

Radiomics predictive models require the input of radiomics features, which must be extracted through a standardized yet intricate preprocessing pipeline (11). This workflow typically includes skull stripping, image registration, downsampling, morphological operations (e.g., mask opening), and other computational steps. While most AIS radiomics studies follow broadly similar preprocessing methodologies, subtle but potentially consequential variations exist across studies in implementation details. The requirement for radiomics feature extraction to strictly adhere to each predictive model’s original preprocessing pipeline introduces several significant challenges.

First, the extraction process itself is inherently complex, involving imaging preprocessing and feature calculation steps (11). Even a single procedure like skull stripping can be performed using various software packages (71–73) or deep learning algorithms (74–76). For example, the commonly used BET (Brain Extraction Tool) offers multiple parameters that yield substantially different results (71). Mastering this technically demanding workflow may prove prohibitively difficult for primary care settings. Furthermore, the complete processing chain is time-intensive, requiring not only execution time for each step but also manual verification and correction. For example, BET-derived masks often require slice-by-slice manual refinement due to suboptimal performance (58).

For a single AIS patient requiring multiple predictive analyses, the necessity to repeat distinct preprocessing pipelines for different prediction models leads to duplicated processing efforts and exponentially increased time consumption. Following preprocessing completion, feature extraction typically relies on packages like PyRadiomics (77), which demands Python programming expertise—while theoretically available in primary care settings, the computational burden becomes non-trivial when extracting thousands of radiomics features. This protracted analytical process fundamentally contradicts the time-sensitive nature of AIS management, where treatment delays measured in minutes may significantly impact clinical outcomes.

Current AIS radiomics research remains prohibitively complex for clinical application in primary care settings, where physicians require simplified predictive tools focused solely on actionable outputs rather than technical processes. Therefore, simplifying AIS radiomics prediction models is a key focus for future development in the field. The ideal solution would involve packaging all the intermediate steps of radiomics into a user-friendly software. In this scenario, doctors would simply input AIS images, select the desired prediction target, and the software would handle the necessary computations, outputting the results directly to the doctor. However, achieving this goal involves overcoming several challenges. (1) implementing automated ROI segmentation to eliminate inter-rater variability and reduce processing time, deep learning neural network is a viable direction (60, 69, 70); (2) establishing standardized preprocessing protocols to enable feature sharing across different prediction models; and (3) consolidating research models into publicly available software tools—or ideally, a unified platform if standardization is achieved. Our review of 80+ existing AIS radiomics studies revealed none have developed such clinical tools, despite their potential to significantly enhance real-world applicability.

## 5 Unreliable prediction results

Given the critical nature of AIS, all clinical assessments—particularly those guiding treatment decisions—must be conducted with utmost precision and reliability. Consequently, radiomics prediction models for AIS must demonstrate high accuracy and robustness to possess meaningful clinical utility. Our systematic analysis of relevant literature reveals that model reliability constitutes a significant concern that cannot be overlooked in current research.

**Figure 5:**
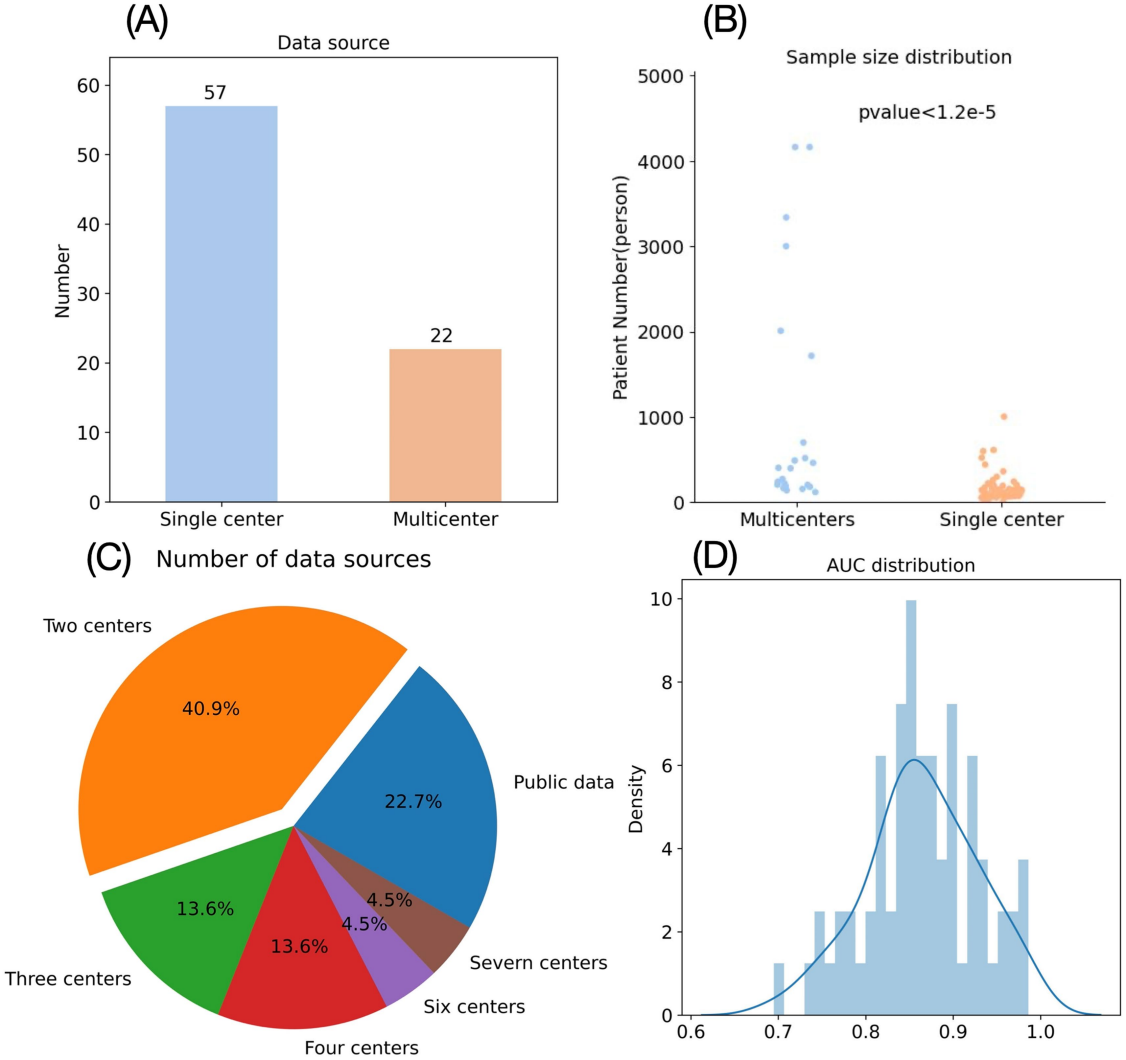
Shortcomings of radiomics research in emergency medicine. **(A)** Data source of AIS radiomics studies. **(B)** Sample number of AIS radiomics studies. **(C)** Distribution of centers for AIS radiomics studies with multicenter data sources. **(D)** Distribution of AUC in AIS radiomics studies.

The use of mutually independent datasets for model training and validation represents a standard approach to assess predictive robustness (78). In our analysis of 78 AIS radiomics studies, we categorized them as single-center (data from one institution) or multi-center (≥2 institutions). Results showed 56 studies (71.8%) utilized single-center data versus only 22 (28.2%) incorporating multi-center data (Figure 5). Among multi-center studies, most involved 2 institutions (60, 69, 70, 79, 80), with 5 leveraging publicly available datasets and a maximum of 7 participating centers (43). Single-institution datasets carry inherent biases that may compromise a model’s generalizability, leaving its predictive performance for data from other institutions uncertain (81). Implementing such unvalidated models in clinical practice—particularly for a critical condition like AIS—requires extreme caution given the potentially grave consequences of unreliable predictions.

In addition to sample diversity, the sample size also significantly impacts a prediction model’s performance and reliability. Generally, larger sample sizes yield more robust predictive models with greater clinical validity (82). Our analysis of 79 AIS radiomics studies revealed substantial variation in sample sizes (Figure 5), ranging from a minimum of 10 patients (48) to a maximum of 4,163 patients (60), with a mean of 420 and median of 191 patients. Notably, only 19 studies (24.3%) exceeded the mean sample size, while 20 studies (25.6%) included fewer than 100 patients. Although some small-sample studies reported impressive predictive performance (AUC > 0.9) (83–85), their reliability remains justifiably questionable given the limited cohort sizes.

Beyond total sample size, the ratio between positive and negative cases warrants careful consideration. Ideally, this ratio should approximate 1:1 (86), but for certain AIS prediction targets with low incidence rates, significant class imbalance often occurs. For example, in Meng et al.’s study predicting hemorrhagic transformation in AIS, the cohort comprised only 71 patients—merely 11 with positive outcomes (87). Although SMOTE algorithm was employed to address this imbalance, this method has inherent limitations: it may introduce redundant information causing overfitting and remains vulnerable to noise interference (88). Notably, despite reporting impressive metrics (AUC = 0.911, accuracy = 0.894) (87), a naive model predicting all negative cases would still achieve 0.845 accuracy in this dataset. This demonstrates that conventional performance metrics (AUC, accuracy, sensitivity, specificity) alone prove insufficient for reliably evaluating radiomics models, particularly for critical conditions like AIS—more comprehensive validation paradigms are essential.

AUC serves as the most widely adopted metric for evaluating model performance, with values exceeding 0.85 typically indicating excellent predictive capability (89). Applying this threshold to our literature analysis—while additionally requiring sample sizes >100 and focusing on “prognosis” and “treatment” prediction categories—yielded only 24 qualifying studies (Figure 5). Among these, 14 utilized CT imaging while 10 employed MRI. From a primary care perspective, CT-based models hold greater practical relevance, prompting further analysis of the 14 CT studies, 1 employed dual-energy CT (a specialized technology rarely available in community settings) (65), 8 focused on endovascular treatment outcomes (largely irrelevant for centers lacking neurointerventional capabilities). This left merely 5 CT-based radiomics models potentially suitable for primary care application—a strikingly small proportion given the extensive AIS radiomics literature. Among these five studies, Ren et al. developed a model for predicting hemorrhagic transformation (HT) in AIS with an AUC of 0.942, accuracy of 0.861, and specificity of 0.949, but sensitivity was only 0.758 (90). Given that HT in AIS patients typically indicates greater severity, higher surgical risks, poorer prognosis, and necessitates closer monitoring plus more conservative treatment approaches (including cautious consideration of endovascular thrombolysis) (91), high sensitivity is clinically paramount for HT prediction models—whereas specificity becomes secondary. The 0.758 sensitivity reported by Ren et al. remains suboptimal for clinical deployment. In contrast, Zhang et al.’s HT prediction model achieved superior sensitivity (0.95) with an AUC of 0.957 and accuracy of 0.861, albeit at the cost of lower specificity (0.75) (92). However, this study’s sample size (*n* = 180) was substantially smaller than Ren et al.’s cohort (*n* = 517), raising concerns about its generalizability.

Consequently, while the quantity of AIS radiomics prediction models appears substantial, rigorous evaluation across critical parameters—including sample size, class distribution, AUC performance, and clinical relevance—reveals that only a limited subset can be considered truly reliable for practical application.

## 6 Discussion

Primary healthcare institutions, as the first line of care for most AIS cases, serve as the frontline for initial diagnosis and treatment, playing a critical role in combating this severe condition. However, it is undeniable that limitations in medical equipment, staffing, and diagnostic capabilities often render these institutions passive in managing AIS patients. Ideally, the widespread adoption of CTP—or even MR perfusion—in primary healthcare settings would be the optimal solution to enhance AIS management. Yet, from a health economics perspective, achieving this remains highly challenging in low-resource regions (93). Even in economically advanced countries like China, comprehensive CTP implementation at the primary level has not yet been realized and cannot be rapidly achieved in the short term (94).

As the frontline providers for most AIS cases, primary care institutions serve as the critical first point of diagnosis and treatment—the essential battleground against this devastating disease (95). However, their diagnostic and therapeutic capabilities remain severely constrained by limited equipment, staffing shortages, and inadequate infrastructure, creating significant challenges in AIS management. Radiomics enables accurate prediction of clinical outcomes through quantitative image feature analysis, offering potential to guide AIS diagnosis and treatment (57). As medical imaging is essential in AIS management, radiomics-based prediction represents a technically feasible approach. The development costs for building precise radiomics models are relatively modest (96), and their deployment in primary care settings would incur negligible expenses compared to the substantial investment required for widespread CTP or MRI implementation. This makes radiomics a potentially cost-effective interim solution to address current diagnostic and therapeutic limitations in resource-constrained settings during the gradual adoption of advanced imaging technologies. However, our critical evaluation demonstrates that most existing AIS radiomics studies exhibit poor clinical applicability for primary care, primarily due to their reliance on sophisticated imaging modalities unavailable in community hospitals and inadequate alignment with frontline clinical decision-making needs.

Current radiomics research in AIS predominantly utilizes CT and MRI in approximately equal proportions. Compared to MRI, CT offers distinct advantages for AIS management, including superior differentiation between ischemic stroke and cerebral hemorrhage, faster examination times, and less restrictive scanning requirements. From both economic and accessibility perspectives, CT remains more widely available in primary care settings, making CT-based radiomics models substantially more feasible for clinical implementation at the community level. This practical consideration strongly suggests that CT-derived models hold greater potential for widespread adoption in resource-constrained healthcare environments.

Nevertheless, half of current AIS radiomics studies rely on MRI—a phenomenon we attribute to the specialized nature of radiomics research. The field’s high technical barriers mean most investigators originate from advanced medical institutions where MRI is routinely available (57). MRI’s multi-sequence protocols and superior resolution enable researchers to extract richer imaging data, theoretically yielding more robust models. While primary care settings demonstrate clear need for CT-based AIS radiomics solutions, these same technical barriers prevent their active participation in model development. This institutional bias manifests in the observed 1:1 CT-to-MRI ratio in published literature.

We must emphasize this analysis does not discount the scientific value of MRI-based radiomics. Should future MRI technology become cost-effective for widespread primary care adoption, such models may indeed prove more clinically valuable. Notably, emerging research explores CT-to-MRI translation using radiomics-guided generative adversarial networks (GANs) (51). Preliminary results demonstrate synthetic MRIs that accurately preserve lesion location and morphology compared to authentic scans. Whether these synthesized images could reliably feed existing MRI-based radiomics pipelines warrants rigorous investigation—an exciting frontier that may eventually bridge the current modality divide.

The mismatch in prediction targets between research and clinical needs stems from systemic differences in healthcare settings, mirroring the previously discussed imaging modality disparity. The observed disparity in radiomics research focus stems fundamentally from the divergent clinical priorities and capabilities across healthcare tiers. Investigators from advanced medical institutions—who dominate radiomics research—naturally extend their scholarly inquiry beyond basic clinical parameters (e.g., symptom onset estimation and complication prediction) to investigate more specialized aspects like functional outcomes, thrombus etiology, and even the temporal stability of radiomics features in AIS. This academic inclination reflects their institutional mandate to pioneer cutting-edge therapies, particularly endovascular interventions that demand sophisticated angiography suites, specialized devices, and highly trained neurointerventional teams (97)—resources typically unavailable in primary care settings. Primary care institutions are typically limited to administering intravenous thrombolysis before promptly transferring AIS patients to advanced centers for potential endovascular therapy evaluation. This fundamental division of clinical responsibilities creates corresponding disparities in research priorities: while frontline providers would benefit most from radiomics models predicting thrombolysis-related outcomes (e.g., hemorrhagic transformation risk or recanalization success), academic researchers at tertiary hospitals naturally focus on endovascular treatment predictions aligned with their institutional capabilities. Our systematic review confirms this misalignment—among identified studies, vascular intervention-related radiomics research (20 publications) outnumbers thrombolysis-focused investigations (2 publications) (43, 44) by an order of magnitude, precisely reflecting the healthcare hierarchy’s influence on scholarly attention.

The issue of unreliable predictive performance persists universally across healthcare settings (98), from primary to tertiary institutions. As previously noted, most AIS radiomics studies utilize single-center datasets, leaving their generalizability to images from different scanners and protocols unverified. Furthermore, many studies suffer from severe class imbalance, necessitating careful scrutiny of their models’ true discriminatory power. Crucially, nearly all existing radiomics research remains retrospective in design—prospective validation would provide more robust evidence for clinical applicability (99). Most fundamentally, we contend that current AIS radiomics models generally lack sufficient predictive accuracy to guide clinical decision-making. Given that therapeutic choices in AIS can profoundly impact survival and long-term outcomes, the demand for exceptionally high model performance cannot be overstated. Yet our analysis reveals only 21 studies (30%) achieved AUCs >0.9, while 10 (14.5%) fell below 0.8. This evidence unequivocally demonstrates the imperative for future research to prioritize substantial improvements in predictive accuracy.

The critical barrier to clinical adoption of radiomics models lies in their failure to transition from research prototypes to practical tools, as nearly all AIS radiomics studies conclude after model validation without developing clinically operable applications. This implementation gap stems from the formidable challenges of integrating the complete radiomics pipeline—from ROI delineation (requiring tools like MRIcron, FreeSurfer or fsleyes) (100–102) through image registration, normalization, and feature extraction (Figure 3)—into a unified platform. We propose establishing standardized preprocessing protocols and a centralized radiomics platform where individual models could be modularly integrated, enabling uniform preprocessing while allowing “plug-and-play” addition of new prediction models—an approach that would eliminate redundant development efforts while ensuring methodological consistency for clinical implementation, particularly crucial for time-sensitive AIS management where prediction reliability directly impacts outcomes.

Is there a way to bypass these cumbersome steps? We believe deep learning approaches like neural networks could provide a solution. Unlike traditional radiomics, neural networks require minimal image preprocessing—raw images can be directly input to generate predictions (103), making them inherently more suitable for tool development. While neural networks operate as “black boxes” (104), this opacity matters little to frontline clinicians who prioritize actionable outputs over methodological transparency. However, the critical limitation remains the substantial training data requirements—individual institutions’ AIS image collections likely prove insufficient, necessitating multicenter public databases to achieve the necessary sample sizes for robust model development (105).

Despite the limitations outlined above, we acknowledge that we remain in the early stages of exploring artificial intelligence in medical imaging. Radiomics simply represents one currently prevalent methodology and does not fully encompass AI’s potential in this field (106). Our critique of current radiomics research stems specifically from the perspective of AIS management in primary care settings, and thus carries inherent limitations. Different diseases present unique characteristics that may reveal new challenges in radiomics applications not addressed here. This paper’s primary objective is to evaluate whether radiomics could serve as a temporary alternative given the current lack of widespread CTP/MRI availability in primary care—not to universally dismiss radiomics or AI development. We recommend future radiomics studies: (1) prioritize NCCT-based approaches, (2) align research objectives with frontline clinical needs, and (3) develop more integrated, user-friendly tools. Crucially, we emphasize that advancing radiomics/AI should never replace efforts to expand CTP/MRI access in community hospitals, nor should it divert focus from investigating AIS etiology and prevention strategies. These fundamental diagnostic capabilities and research directions must remain paramount, with radiomics applications serving only as supplemental tools.

Some argue that future AI research for AIS may not need to address primary care needs, given the emerging “direct transfer” paradigm where suspected AIS patients bypass local hospitals to immediately access comprehensive stroke centers equipped with CTP/MRI and angiographic capabilities, along with multidisciplinary stroke teams (107). This streamlined approach could reduce treatment delays by eliminating unnecessary intermediate evaluations. Should direct referral become standard practice, the transitional utility of radiomics in primary care settings—as proposed in this study—would become obsolete. Consequently, future radiomics research might justifiably exclude primary care considerations. Future AI systems may need to prioritize prehospital severity assessment of AIS patients, safe transfer eligibility evaluation, and integration of multifunctional AI tools. However, implementing direct referral requires robust infrastructure—including accurate clinical triage (108), mobile stroke units (109), teleconsultation (110), and cloud-based imaging sharing—all dependent on regional network capacity, technological resources, and policy frameworks. Global disparities remain profound: While developed nations may achieve nationwide adoption, developing and underserved regions face multifaceted barriers (111). Primary care facilities will retain indispensable roles in AIS management for the foreseeable future. Thus, our investigation into radiomics’ utility in these settings remains clinically relevant during this transitional period.

## 7 Conclusion

In summary, while radiomics could provide valuable decision support for primary care institutions given their current technological and clinical limitations, existing AIS radiomics research has largely overlooked the practical needs of these frontline settings, resulting in limited real-world applicability. To bridge this implementation gap, future studies must prioritize primary care perspectives by optimizing imaging modality selection, aligning clinically relevant prediction targets, ensuring robust model performance meeting frontline diagnostic standards, and developing user-friendly tool—ensuring these advanced analytical methods can truly enhance AIS management at the critical first point of care.
